# Targeting Hippo signaling in cancer: novel perspectives and therapeutic potential

**DOI:** 10.1002/mco2.375

**Published:** 2023-10-03

**Authors:** Liemei Lv, Xiangxiang Zhou

**Affiliations:** ^1^ Department of Hematology Shandong Provincial Hospital Shandong University Jinan Shandong China; ^2^ Department of Hematology Shandong Provincial Hospital Affiliated to Shandong First Medical University Jinan Shandong China; ^3^ Branch of National Clinical Research Center for Hematologic Diseases Jinan Shandong China; ^4^ National Clinical Research Center for Hematologic Diseases the First Affiliated Hospital of Soochow University Suzhou China

**Keywords:** drug resistance, epigenetic, hippo signaling, metabolism, target therapy, tumorigenesis

## Abstract

As highly conserved among diverse species, Hippo signaling pathway regulates various biological processes, including development, cell proliferation, stem cell function, tissue regeneration, homeostasis, and organ size. Studies in the last two decades have provided a good framework for how these fundamental functions of Hippo signaling are tightly regulated by a network with numerous intracellular and extracellular factors. The Hippo signaling pathway, when dysregulated, may lead to a wide variety of diseases, especially cancer. There is growing evidence demonstrating that dysregulated Hippo signaling is closely associated with tumorigenesis, cancer cell invasion, and migration, as well as drug resistance. Therefore, the Hippo pathway is considered an appealing therapeutic target for the treatment of cancer. Promising novel agents targeting the Hippo signaling pathway for cancers have recently emerged. These novel agents have shown antitumor activity in multiple cancer models and demonstrated therapeutic potential for cancer treatment. However, the detailed molecular basis of the Hippo signaling‐driven tumor biology remains undefined. Our review summarizes current advances in understanding the mechanisms by which Hippo signaling drives tumorigenesis and confers drug resistance. We also propose strategies for future preclinical and clinical development to target this pathway.

## Graphical abstract

Hippo signaling consists of conserved kinases tightly regulated by intracellular and extracellular signals. Abnormalities in the Hippo pathway are implicated in a wide range of pathological processes, including tumorigenesis and cancer drug resistance. This review highlights novel strategies to target this pathway and its therapeutic implications, as well as the mechanisms underlying its function in tumorigenesis and cancer drug resistance.

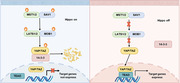

## 1 INTRODUCTION

A coordinated and dynamic process of cell proliferation, differentiation, and apoptosis exerts a vital role in maintaining normal tissue and organ development as well as tissue homeostasis. The dysregulation of this tightly controlled biology may result in a variety of diseases, including cancer. It has been well recognized that Hippo signaling is critical for these biological processes, under normal or disease conditions.^1^, ^2^, ^3^, ^4^ Hippo signaling was originally considered a tumor inhibitory pathway in Drosophila, owing to the overgrowth of tissue caused by gene mutations encoding the key effects.^5^, ^6^, ^7^ In Drosophila and mammals, Hippo signaling is composed of an extremely conserved cascade signaling network modulated by multiple upstream signals.^8^, ^9^ There have been significant efforts to understand how Hippo signaling contributes to development, cell proliferation and differentiation, tissue regeneration and homeostasis, as well as organ size regulation.

It has been well established that the dysregulation of Hippo signaling leads to tumor invasion, migration, disease progression, and cancer drug resistance.^10^, ^11^, ^12^ Thus, targeting Hippo signaling has become a promising strategy in the discovery and development of anticancer drugs. Currently, anticancer agent development targeting Hippo signaling has drawn much attention, with encouraging anticancer effects observed in various tumor models, thus offering profound insights into the role of this pathway in tumorigenesis. However, it has recently been determined that the downstream effectors of Hippo signaling, Yes‐associated protein (YAP) and transcriptional coactivator with PDZ‐binding motif (TAZ), function as tumor suppressors in context‐dependent manners.^13^, ^14^ These findings reiterate the complexity of Hippo signaling in tumorigenesis and explain the potential challenges in therapy development targeting this pathway. Gaining a deeper insight into the multifaceted roles of Hippo signaling in tumors will facilitate the development of novel therapies in both preclinical and clinical settings.

This review intends to provide a thorough and insightful overview of Hippo signaling and summarizes its various upstream modulations that modulate this pathway. In addition, we particularly focus on the functions of Hippo signaling in tumorigenesis and its impact on cancer drug resistance. This review also covers the latest strategies for targeting this pathway as well as the significance of those strategies.

## 2 CORE ELEMENTS OF HIPPO SIGNALING

**FIGURE 1:**
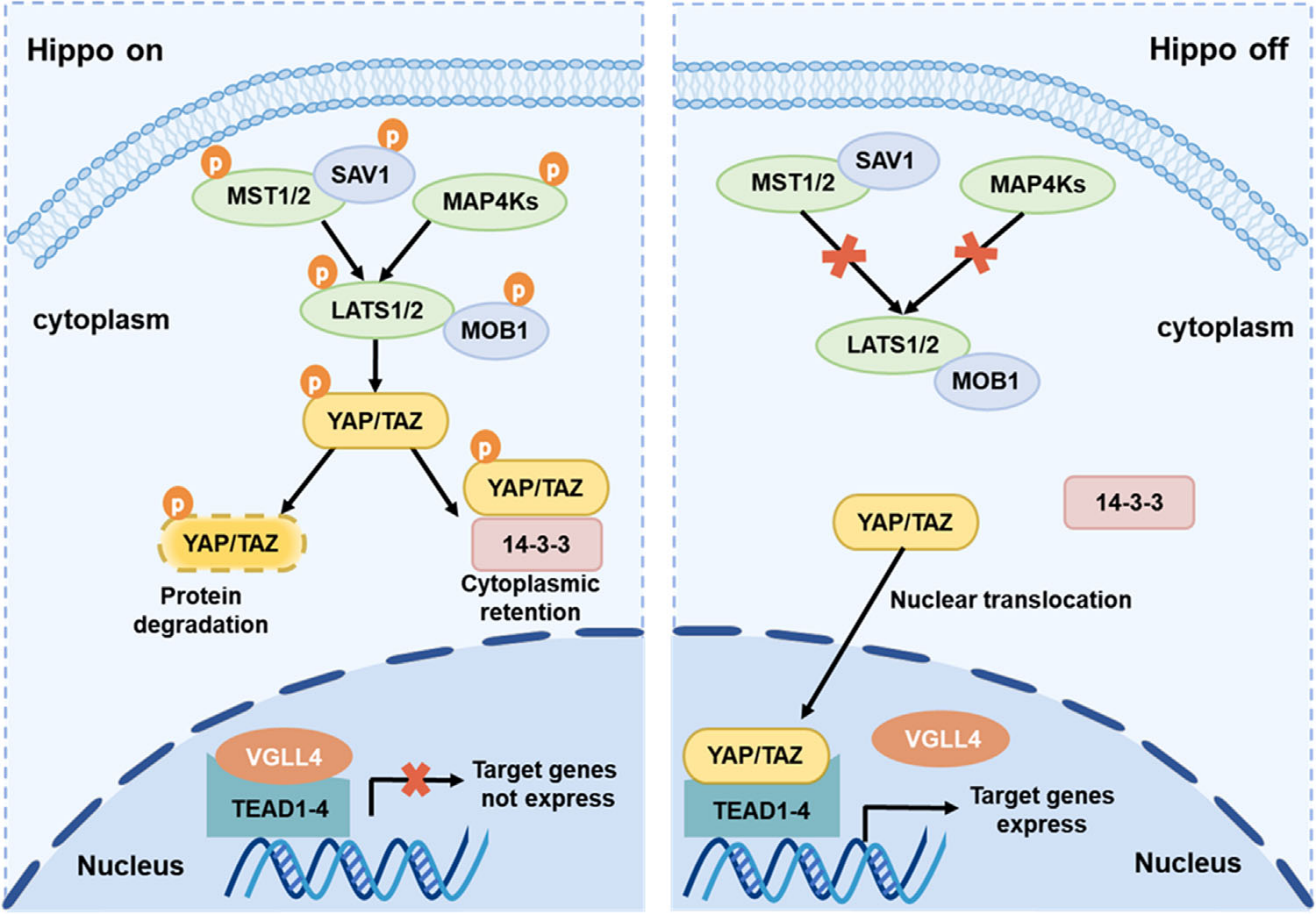
Core elements of the Hippo pathway in mammals. Subcellular localization and transcriptional activity of the transcription coactivators YAP/TAZ are controlled by the Hippo kinase cascade. When the Hippo pathway is ON (Hippo on), YAP/TAZ are phosphorylated by LATS1/2 at multiple sites, bind to scaffold protein 14‐3‐3, and are retained in the cytoplasm. Additionally, the phosphorylation of YAP/TAZ promotes its ubiquitination and degradation. Conversely, when the Hippo pathway is OFF (Hippo off), YAP/TAZ translocate into the nucleus and interact with the TEAD family of transcription factors to promote the transcription of target genes.

The Hippo pathway is composed of a tightly conserved network that ultimately regulates the activity of transcriptional coactivators, YAP and TAZ. In mammals, there are several key proteins in Hippo signaling including mammalian STE20‐like protein kinase (MST1/2), and large tumor suppressor 1/2 (LATS1/2), as well as their respective adaptor proteins salvador homologue 1 (SAV1) and MOB kinase activator 1 (MOB1).^5^, ^6^, ^15^ SAV1, LATS1/2, and MST1/2 are the key molecules of the Hippo pathway (Figure 1). Moreover, some studies suggested that mitogen‐activated protein kinase kinases (MAP4Ks) were also critical elements of Hippo signaling that act in parallel with MST1/2 and can phosphorylate and activate LATS1/2.^16^, ^17^ YAP and TAZ are primary functional exporters of Hippo signaling, performing overlapping and distinctive functions during a wide range of physiological and pathological procedures.^18^

When Hippo signaling is activated, the MST1/2–SAV1 complex phosphorylates and activates the LATS1/2–MOB1 complex, then phosphorylating and inhibiting the activity of YAP and TAZ. Following YAP and TAZ phosphorylation, they bind to scaffold protein 14‐3‐3, remain in the cytoplasm, and are destroyed by the proteasome following ubiquitination. As a result, the activity of YAP and TAZ is suppressed.^19^, ^20^, ^21^, ^22^ Notably, vestigial like family member 4 (VGLL4) was identified as a tumor suppressor that competes with YAP for binding to TEA domain transcription factors (TEADs), thus inhibiting YAP and TAZ transcriptional activity.^23^ Conversely, when the Hippo pathway is inhibited, the phosphorylation cascade will be turned off. Unphosphorylated YAP and TAZ are translocated to the nucleus and interact with TEADs.^24^, ^25^, ^26^ Activated TEADs activate genes responsible for cell proliferation, metastasis, and malignant transformation.^27^ Various biological processes are regulated by the Hippo pathway through this kinase cascade. There is compelling evidence demonstrating that the dysregulated Hippo signaling is implicated in the emergence of a wide range of diseases. A deeper comprehension of the regulatory mechanisms of this pathway and key players may provide a more effective strategy for therapy development and ultimately patient treatment.

## 3 REGULATION OF HIPPO SIGNALING

**FIGURE 2:**
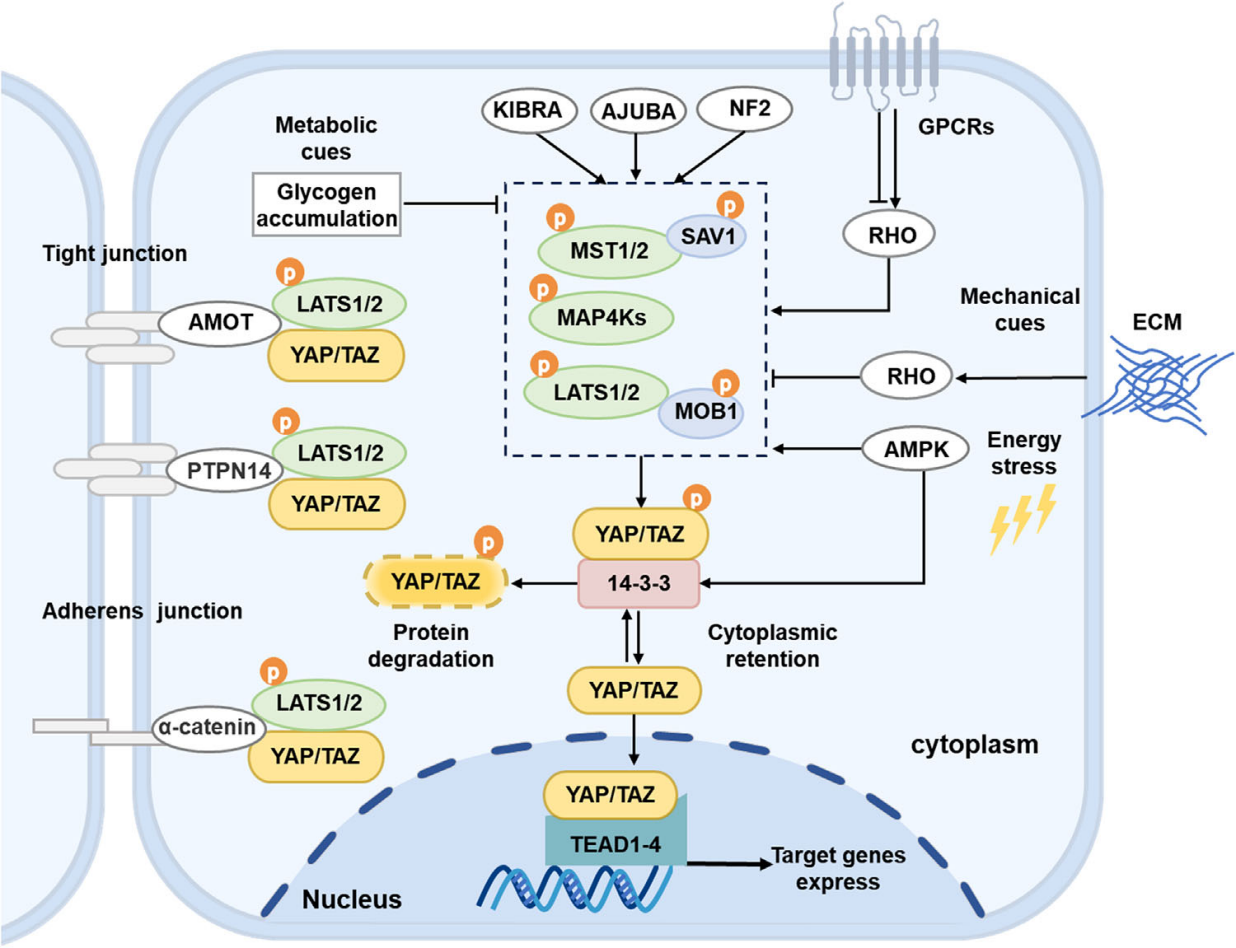
Multiple upstream signals regulate the activity of core components of the Hippo pathway. Metabolic cues such as glycogen accumulation inhibit the Hippo pathway and stimulate YAP, which drives tumorigenesis. There are multiple important upstream regulators of the Hippo pathway, including KIBRA, AJUBA, and NF2. To prevent YAP and TAZ nuclear translocation, AMOT and PTPN14 bind YAP and TAZ at tight junction, while α‐catenin sequesters YAP and TAZ at adherens junction. Mechanical cues, such as extracellular matrix stiffness and cell density, regulate YAP/TAZ activity through Rho GTPases. GPCRs mediate a variety of extracellular signals that activate or inhibit LATS1/2 via Rho GTPases. Cellular energy stress, through glucose starvation‐induced AMPK signaling, by promoting LATS phosphorylation or directly phosphorylating YAP/TAZ.

Previous studies have revealed that YAP and TAZ are major downstream effectors of Hippo signaling. Unlike other signaling pathways, Hippo signaling modulates the phosphorylation‐induced retention and degradation of YAP and TAZ, in response to multiple intracellular and extracellular signals. Several known upstream regulators of Hippo signaling are outlined, including cell polarity, stress signals, mechanical cues, metabolic factors, and soluble factors (Figure 2). These signals affect YAP and TAZ phosphorylation and activity by inhibiting or stimulating LATS1/2 kinases. Notably, dysregulation of these upstream signals has been linked to human malignancy. However, much remains unclear about the upstream factors that regulate this pathway, which needs to be further explored.

### 3.1 Cell polarity

Cell polarity can be described as the distribution of components asymmetrically in a cell, it is typically divided into two types: apical basal polarity (AP) and planar cell polarity. In mammals, several cell junction proteins, including the angiomotin (AMOT) proteins, protein tyrosine phosphatase nonreceptor type 14 (PTPN14), and α‐catenin have been shown to recruit and segregate YAP/TAZ in the cell junction zone by interacting with Hippo signaling components.^28^ AMOT regulates YAP phosphorylation and cytoplasmic segregation by stimulating LATS kinase autophosphorylation.^29^ PTPN14 is an essential negative regulator of the activity of YAP.^30^, ^31^ PTPN14 suppresses cell proliferation by promoting cytoplasmic translocation of YAP in a cell density‐dependent manner.^32^ α‐Catenin works as a tumor suppressor via inhibiting YAP activity and sequestering it within the cytoplasm.^33^ Accordingly, cell polarity and intercellular junctions are essential factors in limiting YAP/TAZ transcriptional activity.

### 3.2 Mechanical cues

Cells are regularly exposed to a variety of biological and mechanical signals from their tissue microenvironment. By responding to these signals, cells are able to regulate their behaviors. Several lines of evidence highlight the close association of Hippo signaling with the mechanotransduction system.^28^ Low cell density and high extracellular matrix stiffness both promote YAP and TAZ accumulation in the nucleus and activate their target genes' transcription.^34^, ^35^ Otherwise, YAP and TAZ are retained in the cytoplasm. Moreover, cell geometry is also involved in Hippo pathway regulation. Round and compact cells retain YAP in their cytoplasm while YAP accumulates in the nucleus in flat spreading cells.^36^ Adhesion kinase‐Src signaling is identified as a negative factor of Hippo signaling that drives nuclear accumulation of YAP.^37^ Moreover, the Hippo pathway is modulated by multiple regulatory proteins such as Rho GTPase, the LIM‐domain protein Ajuba (AJUBA), kidney and brain expressed protein (KIBRA), the tumor suppressor neurofibromin 2 (NF2), α‐catenin, and AMOT.^38^, ^39^, ^40^, ^41^ In summary, different mechanical signals regulate Hippo signaling, and it would be important to explore the mechanisms by which mechanical factors modulate this pathway.

### 3.3 Stress signals

The dynamic control between cellular stress responses and signaling pathways that regulate cell survival and proliferation is crucial to organ growth and tissue homeostasis. Several studies have shown that cellular stress, including energy stress, hypoxia, osmotic stress, or heat stress, participates in Hippo signaling regulation.^42^ As an energy stress sensor, AMP‐activated protein kinase (AMPK) may directly phosphorylate YAP and inhibit YAP transcriptional activity.^43^ Additionally, cellular energy stress blocks YAP activity via AMPK‐dependent LATS kinase activation.^44^ Hypoxia, unlike energy stress, inhibits LATS and induces the activation of TAZ and YAP.^45^ Specifically, hypoxia activates the E3 ubiquitin ligase SIAH2, which facilitates SIAH2 binding with LATS2, subsequently causes the degradation of LATS2, and ultimately leads to YAP activation.^42^ There is evidence for a crosstalk between the HIF pathway and Hippo signaling. It has been reported that osmotic stress induces rapid phosphorylation of YAP‐S128 via NLK kinase, which interferes with YAP's binding to 14‐3‐3, resulting in YAP accumulation in the nucleus and downstream target gene expression.^46^ Osmotic stress also alters the cell membrane distribution of phosphatidylinositol‐4,5‐bisphosphate [PI(4,5)P_2_], leading to NF2 recruitment to the plasma membrane and activation of the Hippo pathway downstream.^47^ Furthermore, osmotic stress triggers p38 activation, which binds directly to TEAD in a Hippo‐independent manner, thus blocking YAP–TEAD‐dependent transcription.^48^ Interestingly, heat stress significantly activates YAP in various cell types and conditions. Specifically, heat shock promotes YAP/TAZ activation by promoting rapid dephosphorylation of LATS and protein ubiquitination, which in turn facilitates cell survival.^49^

### 3.4 Soluble factors

Cellular communication involves not only intercellular junctions and membrane surface molecular contact, but also the transmission of extracellular chemical cues through autocrine or paracrine means. G protein‐coupled receptors (GPCRs) are the most plentiful receptors on eukaryotic cell membranes. There is evidence that a number of extracellular signal‐soluble factors regulate Hippo signaling through GPCRs.^50^, ^51^, ^52^ Depending on which G protein is coupled to the receptor, the activation of GPCR could activate or inhibit the Hippo signaling. Lysophosphatidic acid (LPA) and sphingosine 1‐phosphate (S1P) work via G12/13‐coupled receptors to suppress LATS1/2 activity, which triggers YAP and TAZ activation. On the other hand, glucagon or epinephrine activates Gs‐coupled receptors and then activates LATS1/2, thus inhibiting YAP function.^50^ Furthermore, it has been found that deregulation of GPCR expression or gene mutations is responsible for YAP/TAZ activation and promotes tumor progression.^53^, ^54^ Growth factors, such as insulin‐like growth factor 1 (IGF1) and the epidermal growth factor (EGF) ligand family, can activate receptor tyrosine kinases, resulting in phosphatidylinositol 3‐kinase and AKT activation, disrupting the Hippo kinase complex and thereby increasing YAP1 expression.^55^ These lines of evidence reveal the importance of Hippo signaling in cellular communication and future opportunities for cancer therapy.

### 3.5 Metabolic cues

Hippo signaling regulates extensive biological processes in response to signals from inside and outside the cell, including changes in nutrients and metabolites. This integration of these signals is vital for regulating cell growth and tissue dynamic homeostasis. A variety of connections have been found between the Hippo pathway and the nutrient sensing and metabolic pathways. Furthermore, emerging studies have revealed that a wide variety of metabolic factors affect Hippo signaling, including glucose metabolism,^44^ glutamine metabolism,^56^ and lipid metabolism.^57^

There is abundant evidence that cellular glucose levels are associated with YAP/TAZ activity and subcellular distribution.^58^ Phosphofructokinase (PFK1), a key enzyme of glycolysis, binds TEAD1 in the presence of glycolysis, stabilizing YAP/TAZ and TEAD1, and thus promoting the malignant behavior of breast cancer (BC) cell.^58^ Notably, upon the uptake of glucose, cancer cells store it as an energy source in the form of glycogen. The accumulated glycogen inhibits the Hippo signaling pathway through liquid‐liquid phase separation, activates YAP, and ultimately promotes tumor growth.^59^ Several labs have recently identified the critical features of the hexosamine biosynthesis pathway in regulating metabolic fluxes and O‐GlcNAcylation.^60^, ^61^ O‐GlcNAcylation is also engaged in controlling Hippo signaling. Recent studies revealed that high glucose may link glucose levels to YAP activity via O‐linked β‐N‐acetylglucosamine (O‐GlcNAc) modifications, which in turn regulate Hippo signaling.^60^, ^61^ YAP O‐GlcNAcylation may serve as a potential vulnerability for cancer treatment. Furthermore, the mevalonate pathway regulates BC cell proliferation by stimulating Rho GTPase to inhibit the phosphorylation of YAP/TAZ and accelerate YAP/TAZ nuclear localization.^57^ Hence, understanding the interaction between metabolism cues and Hippo signaling may bring new insights into cancer therapy.

## 4 HIPPO SIGNALING IN TUMORIGENESIS

In a wide range of cancer types, dysregulation of Hippo signaling has been found.^62^, ^63^ Aberrant regulation of this pathway promotes tumorigenesis and confers cancer stem cells with properties such as epithelial–mesenchymal transformation (EMT), drug resistance, self‐renewal, and metastasis.^64^ Therefore, we focus on how this pathway is involved in tumorigenesis in this section.

### 4.1 Dysregulation of Hippo signaling in tumorigenesis

The dysregulation of Hippo signaling has been demonstrated to be prevalent in human malignancies by the Cancer Genome Atlas project through a multiomics analysis of 9125 tumor samples, including mutations, gene fusions, copy number changes, mRNA expression, and DNA methylation.^65^ Several studies indicate that the dysregulation of Hippo signaling is strongly linked to a variety of disease pathologies, including tumorigenesis, making it an appealing target for cancer treatment. For example, it has been demonstrated that YAP is potentially oncogenic in 11q22‐amplified human tumors, and TAZ exerts cancer stem cell‐related characteristics on BC cells.^66^, ^67^ In addition, YAP/TAZ are activated in multiple human tumors including BC, hepatocellular carcinoma (HCC), prostate cancer, and lung cancer,^68^, ^69^, ^70^, ^71^ which is considered to be indicative of poor prognosis. Accordingly, it is imperative to present an integrated understanding of the mechanisms underlying Hippo pathway dysregulation in cancer.

#### 4.1.1 Disruption of Hippo signaling upstream regulators

Although uncommon, loss‐of‐function mutations in YAP and TAZ regulators or activation mutations promoting nuclear YAP and TAZ activity have been identified in multiple cancers. Inactivating mutations in NF2, an upstream regulator of Hippo signaling, have been reported to induce neurofibromatosis type 2 lesions, a genetic disorder linked to unilateral sporadic vestibular nerve sheath tumors and sporadic meningiomas.^72^ NF2 functional mutations are also found in approximately 40% of malignant mesothelioma (MM).^73^ Interestingly, about 45% of MM cases have been observed with genetic mutations in LATS2, an important signal transducer in the Hippo pathway.^74^ Furthermore, the deletion of YAP inhibits NF2 knockout‐induced tumorigenesis in mice, highlighting that YAP is a significant effector of NF2 in the regulation of growth.^75^ These studies suggest that YAP could be an appealing therapeutic target for neurofibromatosis 2.

Aberrant GPCR signaling is implicated in a number of human diseases, notably cancer. The GNAQ and GNA11 genes, which encode the heterotrimeric G proteins Gq and Gα11, respectively, are mutated in almost 80−90% of uveal melanomas, which was shown to drive nuclear YAP activation and was believed to promote tumorigenesis.^53^, ^76^, ^77^ Moreover, the insulin‐like growth factor‐1 receptor (IGF‐1R) is involved in the regulation of Hippo–YAP signaling in diffuse large B‐cell lymphoma (DLBCL) tumorigenesis.^78^ The inhibition of IGF‐1R leads to dysregulation of Hippo signaling activation. An in‐depth study of these changes could help shed light on how cancer treatments could be developed.

#### 4.1.2 Gene fusions in tumors associated with Hippo signaling

Fusion genes are significant drivers of tumorigenesis and development, usually involving gene expression change or/and changes in their activities. Epithelioid hemangioendothelioma (EHE) refers to an unusual malignant vascular tumor characterized by TAZ (3q25) and CAMTA1 (1p36) participating in t (1;3) (p36; q25) chromosomal translocations.^79^ The fusion of TAZ and CAMTA1 (TC) causes nuclear localization of the two proteins and homeodomain activation, which triggers TAZ‐like transactivation and tumorigenic activity in TC‐expressing cells.^80^ Targeting TAZ and TC chimeric proteins may be a promising therapeutic strategy for EHE. Further, there was another fusion of YAP1 with TFE3 found in a small proportion of EHE cases.^81^, ^82^ In fact, fusions of multiple crucial members of Hippo signaling were detected in lung cancer, including LATS1, YAP, TAZ, TAOK1, TAOK3, FAT1, and PTPN14.^81^ Thus, these findings strongly support that fusion genes in Hippo signaling can act as potential cancer drivers.

#### 4.1.3 Elevated YAP/TAZ levels and activity in tumors

Research over the past two decades has generally accepted the view that YAP and TAZ act as pro‐oncogenes, whereas Hippo cascade kinases are tumor suppressors. It is commonly observed that YAP and TAZ are activated in human malignancies and are critical to the development or growth of various cancers.^27^, ^83^, ^84^ It should be noted, however, that YAP and TAZ are not considered oncogenes, and their activation or overexpression is insufficient to initiate cancer. YAP and TAZ induce tumorigenesis in response to or in association with oncogenic conditions.^85^ Nevertheless, the elevated nucleus expression of YAP and TAZ proteins is closely linked to adverse outcomes and increased drug resistance in multiple tumors.^27^ This evidence supports the critical role of YAP and TAZ in tumor development. Targeting YAP and TAZ in tumors with high levels of expression or activity of these proteins could be an attractive strategy.

### 4.2 Crosstalk with other signaling pathways

Cross‐talk between Hippo signaling and other signaling pathways is considered a prominent element in the integration of cell growth and fate decisions. It has been observed that diverse signaling pathways regulate or cross‐talk with Hippo signaling.^86^ For example, Hippo signaling can positively and negatively regulate the Wnt pathway by interacting with Wnt/β‐catenin signaling transduction in several different ways.^18^ The Hippo pathway suppresses Wnt/β‐catenin signaling transduction by facilitating the interaction between TAZ and Dishevelled.^87^ Activation of Wnt/β‐catenin signaling inhibits the formation of HCC via suppressing the positive feedback loop between YAP/TAZ and Notch signaling.^88^ Overall, these studies indicate that Wnt signaling could regulate YAP and TAZ, which in turn are controlled by YAP and TAZ.

Additionally, Hippo signaling might be crucial in reversing the effects of transforming growth factor‐beta (TGF‐β) signaling in tumor cells at the early and late stages. Recent studies have identified TGF‐β receptor 2 (TGFBR2) as a tumor suppressor with unique prognostic value in early‐stage non‐small cell lung cancer (NSCLC).^89^ TGFBR2 is inhibited simultaneously by YAP/TAZ via two mechanisms: post‐transcriptionally via the miR‐106b‐25 cluster and transcriptionally through engagement with the EZH2 epigenetic repressor.^89^ An innovative therapeutic approach for treating NSCLC may involve cotargeting of YAP/TAZ and EZH2. Interestingly, cytoplasmic YAP/TAZ was found to induce SMAD activity in the nucleus, whereas nuclear YAP/TAZ inhibits the accumulation of SMAD in the cytoplasm.^90^ Low cell density‐induced activation of TGF‐β‐SMAD is dependent on nuclear YAP/TAZ, while YAP/TAZ‐induced stem cell self‐renewal is dependent on nuclear SMAD.^91^ Recent studies suggest that the Hippo signaling works closely with other signaling pathways in tumor cells, including the AMPK pathway,^43^ KRAS signaling,^92^ and MAPK/ERK signaling to drive tumorigenesis.^93^ These pathways have been clinically validated to contribute significantly to the development and progression of tumors. However, it still needs to be further defined how the Hippo signaling works in concert with them.

### 4.3 Hippo signaling in cancer metabolic reprogramming

**FIGURE 3:**
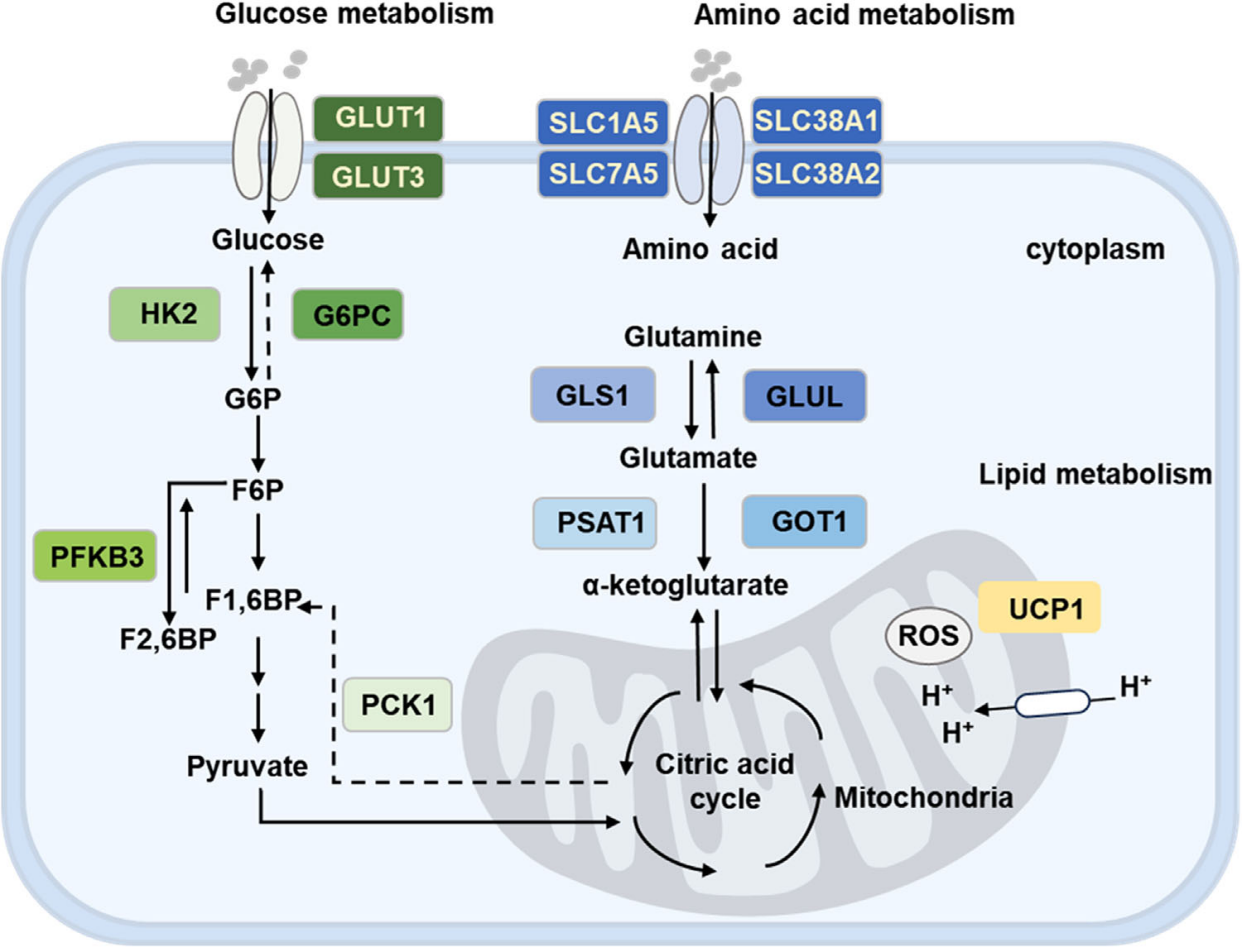
The Hippo pathway regulates cellular metabolic reprogramming. The Hippo pathway regulates glucose metabolism by increasing glucose uptake and glycolysis. YAP and TAZ modulate glucose metabolism by promoting the expression levels of glycolysis‐related enzymes (GLUT1, GLUT3, HK2, and PFKB3), and also by repressing the transcription of gluconeogenesis key enzymes, such as G6PC and PCK1. Furthermore, YAP and TAZ can affect amino acid uptake by upregulating the expression of amino acid transporters (SLC1A5, SLC7A5, SLC38A1, and SLC38A2). As well, YAP and TAZ regulate glutamine metabolism in a context‐dependent manner through GLS1, GLUL, GOT1, and PSAT1. The Hippo pathway also plays a vital role in fatty acid metabolism. UCP1 expression is regulated by YAP and TAZ, which promote lipid oxidation and reduce lipid accumulation.

Metabolic reprogramming is one of the hallmarks of tumor cells.^94^ Tumor cells need to consume large amounts of glucose, amino acids, and fatty acids to meet their unrestricted growth and proliferation.^61^, ^95^, ^96^ Increasing evidence suggests that the Hippo pathway regulates metabolic reprogramming to facilitate the growth of malignant tumors. For example, the Hippo–YAP pathway promotes tumor progression by reprogramming glucose metabolism.^97^ Despite being an emerging field, extensive studies suggest that Hippo signaling controls a variety of metabolic processes (Figure 3). Understanding the new Hippo‐regulated metabolism could reveal vulnerabilities to be exploited in future therapies.

#### 4.3.1 Glucose metabolism

Unlike normal cells, tumor cells heavily rely on aerobic glycolysis to fuel their rapid proliferation.^98^ It has been demonstrated that TAZ and YAP play an important role in regulating the growth and survival of tumor cells, as well as controlling cancer metabolism.^99^ Research indicates that YAP regulates the expression of glucose transporter protein 1 (GLUT1), which in turn regulates glucose metabolism.^100^ Hypoxic stress facilitates the binding of YAP to HIF‐1α in the nucleus as well as keeps the stability of the HIF‐1α protein. The YAP/HIF‐1α complex activates the transcription of GLUT1, which accelerates glycolysis in tumor cells as a result.^97^ The GLUT3, which is frequently overexpressed in cancers, is a direct transcriptional target of the PAK4–YAP/TAZ signaling axis.^101^ The dependency of GLUT3 for glucose uptake and survival makes these tumor cells susceptible to YAP inhibition. Moreover, decreased glycolysis or impaired glucose metabolism reduces the transcriptional activity of YAP/TAZ.^58^ Zheng et al.^102^ identified hexokinase 2 (HK2) and phosphofructokinase B3 (PFKB3) as indirect transcriptional targets of YAP. Moreover, peroxisome proliferator‐activated receptor gamma coactivator and gluconeogenic genes including glucose‐6‐phosphatase catalytic subunit (G6PC) and phosphoenolpyruvate carboxykinase 1 (PCK1), were also identified as important targets of YAP.^103^ These studies highlight the significant contribution of YAP to glucose metabolism, possibly via metabolic reprogramming to the anabolic requirements of cells to support growth.

#### 4.3.2 Amino acid metabolism

Glutamine is the most abundant amino acid in the body. Reprogramming of glutamine metabolism is one of the hallmarks of cancers. Increasing studies suggest that YAP is essential for glutamine metabolism.^104^, ^105^, ^106^ It has been reported that YAP increases the expression of glutamine synthetase (GLUL), which subsequently improves glutamine levels and enhances the synthesis of de novo nucleotides. YAP‐dependent GLUL regulation is indispensable for human HCC cell growth, and inhibitors of YAP or glutamine synthetase inhibit HCC cell growth.^104^ Further evidence indicates that Hippo signaling is implicated in glutamine metabolism regulation. For example, recent studies reveal that YAP induces expression of the amino acid transporter protein SLC38A1, thus activating mTORC1 and promoting tumor growth.^107^ Yang et al.^56^ revealed that YAP/TAZ promotes the expression of transaminases GOT1 and PSAT1. Moreover, transaminase inhibitor aminoacetate inhibits BC cell growth in a YAP/TAZ‐dependent manner. Oncogenic KRAS mutations upregulate amino acid transporters (SLC7A5, SLC1A5, and SLC38A2) expression via YAP, promoting the activation of mTOR and proliferation of CRC cells.^108^

It should be mentioned that YAP and TAZ might influence the uptake of amino acids, including SLC1A5, SLC7A5, and SLC38A1.^109^, ^110^, ^111^ When amino acid transporters are at low concentrations, these high‐affinity transporters are crucial for amino acid uptake. In summary, active YAP and TAZ promote cellular amino acid metabolism via inducing metabolic enzymes, as well as enhancing the ability of cells to take up amino acids for rapid growth.

#### 4.3.3 Lipid metabolism

To meet the energy requirements for the unrestricted proliferation of tumor cells, fatty acid metabolism also plays a prominent part in tumor metabolism. It has been confirmed that the inactivation of Hippo signaling promotes lipid accumulation.^112^ In PTEN knockout mice, SAV1 knockdown accelerated steatosis and activated YAP/TAZ while YAP and TAZ deletion prevented PTEN knockout‐induced steatosis.^112^ This evidence highlights the significance of Hippo signaling in lipid metabolism. Furthermore, the downregulation of LATS2 in HCC cells leads to SREBP activation and excess cholesterol accumulation.^113^ Curiously, Tharp et al.^114^ found that YAP and TAZ reduce fat deposition and drive adipocyte thermogenic activity in brown adipocytes by controlling transcriptional uncoupling protein 1 (UCP1) expression. A recent investigation has revealed that YAP‐driven activation of adipogenesis relies on mTORC1 activation by serum‐ and glucocorticoid‐regulated kinase 1 and stimulation of the SREBP‐regulated transcriptional program.^115^ It has been uncovered that de novo lipogenesis represents a metabolic weakness. The blockage of YAP activity may be a promising approach to treating cancer. So, the regulation of metabolism by Hippo signaling could be exploited for anticancer therapy development. For example, dropwort, an organic compound, inhibits YAP/TAZ/TEAD oncogenic signaling in mesothelioma by inducing metabolic reprogramming.^116^ Together, these findings provide new insights into Hippo signaling in cancer metabolism, however, much work is still needed in elucidating the molecular mechanisms by which Hippo signaling affects metabolic changes to take full advantage of targeting this pathway in therapy development.

### 4.4 Hippo signaling in epigenetic modulation

Epigenetic hallmarks are closely associated with tumor development, and they regulate gene function and expression levels, primarily via DNA methylation, histone modifications, noncoding RNA regulation, and chromatin structural remodeling.^117^ The Hippo pathway, a well‐defined functional pathway that controls tumor development and progression, is an important part of the epigenetic machinery.

#### 4.4.1 DNA methylation

It has been reported that the extensive components of Hippo signaling are also tightly regulated by various epigenetic signals, leading to altered cancer phenotypes.^118^, ^119^ The microtestoid (MORC) protein family are critical epigenetic modifiers and readers that regulate the Hippo pathway through DNA methylation. MORC2 forms a complex with DNA methyltransferase 2A at the promoters of NF3 and KIBRA, resulting in its DNA hypermethylation and transcriptional repression, promoting cancer stemness and tumorigenesis.^120^ A scaffold protein of Hippo signaling, RASSF1A, is usually epigenetically silenced in tumors, resulting in the emergence of various pathological processes, including tumorigenesis.^121^ Frequent hypermethylation and silencing of gene expression of KIBRA were found in pediatric acute lymphoblastic leukemia cell lines.^122^ Further, ZH2‐mediated modification of H3K27me3 and DNA methylation are the underlying causes of MST1 silencing.^123^

#### 4.4.2 Noncoding RNA regulation

Exploring the crosstalk between long‐stranded noncoding RNAs (lncRNAs) and Hippo signaling in tumor progression has recently become an attractive area of extensive research. It has been demonstrated that the crosstalk between lncRNA and the Hippo signaling assists in cancer development and progression.^124^, ^125^, ^126^ lncRNA GAS5 inhibits colorectal cancer progression by interacting with and enhancing the phosphorylation and degradation of YAP in osteosarcomas.^124^ Upregulation of MIR100HG promotes osteosarcoma progression via epigenetic silencing of LATS1 and LATS2 and inactivating Hippo signaling.^125^ Overexpression of lncRNA TNRC6C‐AS1 promotes thyroid cancer progression by blocking Hippo signaling and promoting the methylation of STK4 in thyroid cells.^126^ LinC00673 likely functions as a sponge for miR‐515‐5p to upregulate MARK4 and blocks the Hippo pathway for oncogenic effects.^127^ Interestingly, TEAD4‐mediated transcriptional activation of MNX1‐AS1 regulates gallbladder cancer progression by continuously inhibiting Hippo signaling via a positive feedback loop.^128^ Moreover, lncRNA, RP11‐616M22, contributes to GIST resistance to imatinib via interaction with RASSF1.^129^ Notably, small lncRNAs may be involved in the crosstalk of Hippo signaling in cancer progression. In particular, miR‐152 and miR‐497 may affect Hippo signaling through RASSF1A.^130^ The upregulation of miR‐135B inhibits the LZTS1 and Hippo signaling, thereby stimulating cancer progression and stemness.^131^

#### 4.4.3 Histone modifications

Emerging evidence indicates that the dysregulation of histone modifications, including methylation, acetylation, and phosphorylation, is closely associated with a wide range of diseases, including cancers.^132^ The Hippo pathway is also an important component of histone modifications. Growing evidence has also revealed the significance of phosphorylation in Hippo signaling.^18^ Not surprisingly, acetylation in connection with Hippo signaling plays a different role in tumorigenesis. Jin et al.^133^ found that oxidative stress inhibits the degradation of acetylated acetyltransferase CBP to promote MOB1 acetylation and activate the Hippo signaling pathway. However, acetylation of LATS1 blocked Hippo signaling, converting LATS1 from a tumor suppressor to a tumor promoter.^134^

It has also been demonstrated that Hippo signaling is regulated by O‐GlcNAcylation. In the presence of abundant extracellular glucose, O‐GlcNAcylation of YAP at its serine 109 sites directly hinders its interaction with LATS1 and promotes its transcription.^60^ TET1, a 5‐methylcytosine dioxygenase, is a direct target of YAP and a key regulator of genome‐wide epigenetic and transcriptional reprogramming. Mechanistically, activating YAP leads to TET1 expression, which interacts with TEAD, leading to regional DNA demethylation, histone H3K27 acetylation, and chromatin opening of YAP target genes, thus facilitating transcriptional activation.^135^

Collectively, there is compelling evidence that Hippo signaling is controlled by epigenetic mechanisms, which play an integral role in tumorigenesis. Several anticancer drugs targeting epigenetics, such as DNA methyltransferase inhibitors and histone deacetylase inhibitors, have shown antitumor activity. Targeting epigenetic regulation in the Hippo pathway could be a promising strategy for individualized cancer treatment development.

## 5 TARGETING THERAPY OF HIPPO SIGNALING

**FIGURE 4:**
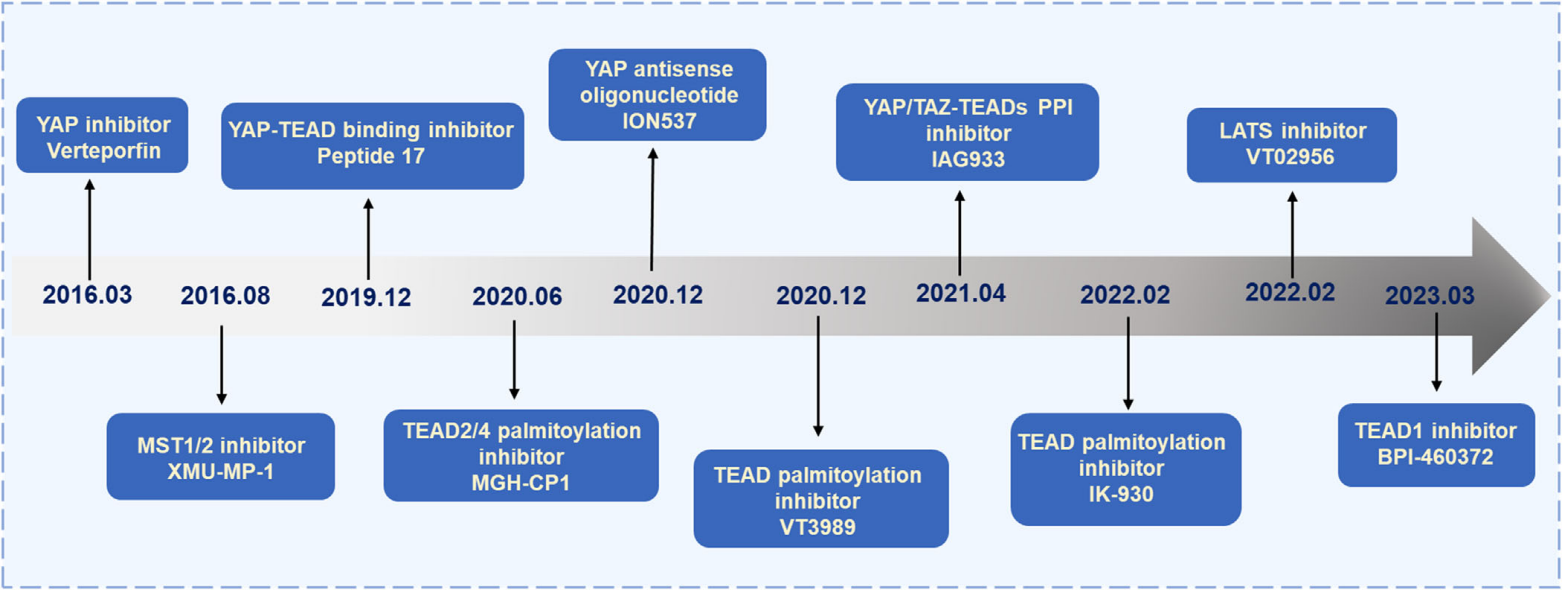
Hallmarks of diverse categories of Hippo pathway targeted agents.

Accumulating research has demonstrated that the key components of Hippo signaling are significantly linked with numerous diseases.^136^, ^137^, ^138^ YAP and TAZ, the transcriptional coactivators with aberrant activation commonly observed in human malignancies, play multifaceted roles in tumorigenesis, migration, and drug resistance. Tumor treatment strategies targeting the Hippo pathway are believed to have favorable prospects for clinical translation.^63^, ^139^ Recent years have seen the emergence of a wide range of small‐molecule inhibitors targeting the Hippo pathway (Figure 4), some of which have already been enrolled in clinical trials while others are undergoing preclinical testing (Table 1). Thus, we focus on compounds currently in preclinical and clinical development that modulate or block the Hippo signaling pathway and hope these compounds will provide patients with new treatment options.

**TABLE 1:** Recent clinical trials and preclinical evaluations on Hippo pathway targeted agents in tumors.

Compound (sponsor)	Mechanism	Administration	Phase	Condition or disease	Trial number
VT3989 (Vivace Therapeutics)	TEAD palmitoylation inhibition	Oral	I	Solid Tumor Mesothelioma	NCT04665206
IK‐930 (Ikena Oncology)	TEAD palmitoylation inhibition	Oral	I	Solid tumors Mesothelioma Epithelioid hemangioendothelioma NF2 deficiency YAP1 or TAZ Gene Fusions	NCT05228015
ION537 (Ionis Pharmaceuticals)	YAP antisense oligonucleotide	Intravenous injection	I	Advanced solid tumors	NCT04659096
IAG933 (Novartis)	YAP/TAZ‐TEADs PPI inhibitor	Oral	I	Mesothelioma	NCT04857372
BPI‐460372 (Betta Pharmaceuticals Co., Ltd.)	TEAD1 inhibitor	Oral	I	Advanced solid tumor	NCT05789602
XMU‐MP‐1	MST1/2 inhibitor	–	Preclinical	Protest cancer Breast cancer	–
SBP‐3264	MST1/2 inhibitor	–	Preclinical	Acute myeloid leukemia	–
VT02956	LATS inhibitor	–	Preclinical	Breast cancer	–
CA3	YAP inhibitor	–	Preclinical	Esophageal adenocarcinoma Osteosarcoma tumor pancreatic cancer	–
Verteporfin	YAP inhibitor	–	Preclinical	Glioblastoma Breast cancer Hepatocellular carcinoma	–
VGLL4 peptide mimic	YAP–TEAD interaction inhibitor	–	Preclinical	Gastric cancer Colorectal cancer	–
Peptide 17	YAP–TEAD inhibitor	–	Preclinical	Ovarian cancer	–
MYF‐01‐37	TEAD inhibitor	–	Preclinical	NSCLC	–
Flufenamic acid derivative	TEAD inhibitor	–	Preclinical	Glioblastoma	–
MGH‐CP1	TEAD2/4 inhibitor	Oral	Preclinical	Nasopharyngeal carcinoma	–

PPI, protein–protein interaction.

### 5.1 Targeting core kinases of Hippo signaling

A great deal of effort has been invested in identifying and discovering inhibitors of Hippo core kinase activity. There are several types of Hippo core kinase inhibitors, but the most common are MST kinase activity inhibitors. Increasing research has shown that XMU‐MP‐1, an inhibitor of MST kinase, plays an essential part in tissue repair and regeneration.^140^, ^141^ This compound, for example, is effective in improving cardiac hypertrophy,^142^ chronic and acute liver injury,^140^ as well as acute liver failure.^143^ Interestingly, a 2‐month dose of XMU‐MP‐1 daily was not found to induce tumors in control mice.^140^ New insights into cancer treatment were also provided by this study. That is, the inhibition of these kinases may be effective in the short term. Recently, a selective small molecule inhibitor, compound 20 (SBP‐3264), has been shown promising therapeutic potential in the treatment of acute leukemia.^144^ It was found that SBP‐3264 and BCL‐2 inhibitor venetoclax inhibited the proliferation of AML cells in vitro synergistically.

Moreover, compelling evidence demonstrated an unexpected but promising role for LATS1/2 in immunotherapeutic strategies for Hippo‐independent tumors, highlighting the possibility of designing LATS1/2 kinase inhibitors for enhancing antitumor immunity.^145^ For this reason, VT02956, an inhibitor of LATS, is an attractive candidate for therapy development. Recent studies confirmed that VT02956 inhibits ESR1 expression and suppresses the proliferation of ER (+) BC cells.^146^ In brief, targeting Hippo core kinase activity in cancer therapy represents a promising therapeutic strategy. However, it still requires considerable efforts to determine the benefits vs risks of such inhibitors as they are being advanced into clinical studies.

### 5.2 Targeting YAP and TAZ activity

YAP/TAZ is regulated by upstream signals of the Hippo pathway. Regulation of YAP/TAZ activity and subcellular localization is the goal of small molecule drugs. YAP activation has been found to increase resistance to RAF and MEK inhibitors,^147^ so targeting YAP may offer a new cancer treatment approach.^146^ A novel small molecule YAP inhibitor, CA3, has demonstrated the potential to inhibit tumor growth in multiple cancers, including esophageal adenocarcinoma, pancreatic cancer, and osteosarcoma.^148^, ^149^, ^150^ Depletion of YAP and TAZ by knockdown or genetic knockout to inhibit tumor cell proliferation has been shown in various cancer cell lines and animal models.^78^, ^151^ A promising therapeutic approach to combating cancer may involve blocking YAP and TAZ. Oligonucleotide‐based therapies such as siRNA therapy are viable options in anticancer treatment development as it often offers higher specificity compared with some small molecule inhibitors. The antisense drug ION1537 is presently being examined in phase I clinical trials for the treatment of advanced solid tumors.^152^ It is, however, much more difficult to deliver antisense RNA than conventional small‐molecule drugs. Fortunately, siRNA‐lipid nanoparticle (siRNA‐LNP) technology may help address this challenge.^153^ It is noteworthy that recent studies have demonstrated that SuperHippo, a WWC‐derived designer protein, significantly inhibits tumorigenesis in genetically engineered mouse models of HCC. Expression of the SuperHippo minigene distinctly activates LATS1/2 activity, leading to complete inactivation of YAP/TAZ and effective inhibition of tumorigenesis in multiple tumor models.^154^ These findings provide potentially exciting possibilities for the future therapy of cancer patients.

### 5.3 Targeting YAP–TEAD/TEAD

Transcription factors are usually not considered druggable,^155^ but TEADs are the exception. TEADs have a hydrophobic pocket and possess enzymatic activity, making them an attractive target for small‐molecule drugs. Recent research has found that targeting the YAP/TAZ–TEAD transcriptional complex is an attractive strategy.^156^ Verteporfin (VP), a compound that blocks the interaction between YAP and TEAD, was identified from a small library of United States Food and Drug Administration (US FDA)‐approved compounds.^157^, ^158^ VP suppresses tumor proliferation by blocking the YAP and TEAD interaction,^159^ but this effect may not be limited to the YAP–TEAD complex. Additionally, some studies have demonstrated that VP is proteotoxic and lacks specificity,^160^ which limits its application in the clinic. Nevertheless, inhibitors of YAP–TEAD/TEAD, including MYF‐01‐37,^161^ VGLL4 peptide mimics,^23^ peptide 17,^162^ and MGH‐CP1, are still being explored.^163^ Unlike others in the group, MYF‐01‐37 has since been identified as a covalent binding agent for TEAD, which effectively targets NSCLC cells with EGFR mutations.^161^ Regarding the mechanism, VGLL4 peptide mimetics compete with YAP binding to TEAD via the TDU domain, thus functioning as a YAP antagonist.^23^ The MGH‐CP1, an inhibitor of auto‐palmitoylation of TEAD2 and TEAD4, inhibits nasopharyngeal carcinoma cell migration, invasion, and cisplatin resistance.^163^ In addition, work by Pobbati et al.^164^ found that a nonsteroidal anti‐inflammatory drug (NSAID), flufenamic acid, inhibits TEAD function and TEAD–YAP‐dependent processes by binding to the deep hydrophobic pocket of TEAD. These studies provide a theoretical basis for the idea that TEAD transcription factors can be targeted by small‐molecule drugs.

Studies have confirmed that TEAD's oncogenic activity is exerted through its palmitoylation,^165^, ^166^ thereby providing an avenue for inhibitor development targeting palmitoylation. Utilizing computer modeling and chemical library screening, a number of TEAD inhibitors were identified.^156^, ^167^, ^168^ These inhibitors block palmitoylation of TEAD and expression of TEAD target genes and have shown strong anticancer activity in cell lines and animal models. Currently, the TEAD palmitoylation inhibitors VT3989 (NCT04665206), IK‐930 (NCT05228015), and TEAD1 inhibitor BPI‐460372 (NCT05789602) are in phase I clinical trials. In addition, recently published phase I clinical trial results showed that VT3989 has a good safety profile and tolerability, and demonstrated anticancer activity signal.^169^ Taken together, targeting Hippo signaling has emerged as an appealing cancer treatment strategy.

### 5.4 Natural agents

It is worth noting that several natural plant‐derived phytochemicals exert unexpected antitumor activity by modulating the Hippo pathway.^170^ Naturally, natural agents targeting the Hippo pathway have received considerable attention. Ursolic acid (UA), a botanical herbal compound, has shown impressive anticancer efficacy in various types of tumors.^171^, ^172^, ^173^ UA inhibits gastric cancer (GC) cell proliferation and metastasis through activating Hippo signaling via RASSF1 and exhibits antitumor activity.^174^ It has been suggested that Pinus koraiensis pinecones (PEO), an herbal preparation, might exhibit antitumor activity by Hippo signaling.^175^ Liquiritigenin, a natural flavonoid isolated from licorice, is both a hepatoprotective agent and stimulates LATS to induce phosphorylation of YAP and suppress tumor cell growth.^176^ Decursin and Marsdenia tenacissimae extract can pathway inactivate YAP to exert antitumor effects and are potential candidates for the treatment of YAP‐dependent tumors.^177^, ^178^ Matrine, an alkaloid present in Sophora flavescens Ait, activates MIEF1‐associated mitochondrial division through the LATS2‐Hippo pathway, inhibiting tumor cell survival.^179^ Tanshinone IIA (Tan IIA) is a tanshin derivative that induces apoptosis in colorectal cancer cells by promoting mitochondrial fission and stimulating the Mst1‐Hippo signaling.^180^ A recently discovered curcumin derivative has also been reported to exert antitumor properties by inducing apoptosis in GC cells through activation of Hippo signaling.^181^ The effectiveness of these natural phytochemicals in targeting Hippo pathway‐dependent tumor cell growth shows definite therapeutic promise for future cancer treatment.

## 6 HIPPO PATHWAY IN CANCER DRUG RESISTANCE

Significant progress has been made in treating patients with various types of cancer due to the application of novel antitumor drugs. Due to primary or acquired resistance, large numbers of patients with malignant tumors are unable to achieve the ideal therapeutic effect. Cancer patients still suffer from drug resistance, which limits their prognosis.^182^ A growing body of research supports that Hippo signaling makes a vital contribution to cancer resistance to therapy. In fact, key molecular components of the Hippo pathway have been identified as being related to cancer drug resistance.^183^ For instance, YAP regulates trametinib resistance in head and neck squamous cell carcinoma (HNSCC)^184^ and confers resistance to 5‐FU and docetaxcel in esophageal cancer.^185^ Interferon‐γ triggers tumor resistance to PD‐1 immunotherapy and is enhanced by YAP activation.^186^ As a comprehensive study of the molecular mechanism between Hippo signaling and cancer drug resistance would be beneficial to deal with drug resistance issues, we here discuss in detail how the vital elements of this pathway contribute to cancer drug resistance (Table 2).

**TABLE 2:** Summary of Hippo signaling elements implicated in cancer drug resistance in recent years.

Cancer type	Components	Related drug resistance	References
Nasopharyngeal carcinoma	TEAD	Cisplatin	^163^
Head and neck squamous cell carcinomas	YAP	Trametinib	^184^
Lung cancer	YAP, TAZ	Erlotinib, EGFR‐TKIs, osimertinib, cisplatin	^187^, ^188^, ^189^, ^190^, ^191^
Esophageal cancer	YAP	5‐FU, docetaxcel	^185^
Gastrointestinal stromal tumor	RASSF1	Imatinib	^129^
Colorectal cancer	YAP, TAZ	Cetuximab, 5‐FU	^192^, ^193^, ^194^
Hepatocellular carcinoma	YAP	Adriamycin, sorafenib	^195^, ^196^
Pancreatic cancer	MST1, LATS2, MOB1, SAV1, YAP	Gemcitabine	^197^, ^198^
Prostate cancer	YAP	Docetaxel	^199^
Bladder cancer	RASSF1A	Cisplatin, doxorubicin	^200^
Breast cancer	YAP, TAZ, TEAD	Paclitaxel, doxorubicin, lapatinib, tamoxifen, mitomycin C, cisplatin, etoposide	^201^, ^202^, ^203^, ^204^, ^205^
Ovarian cancer	YAP, MST1, SAV1, TEAD	Cisplatin, taxol, bleomycin	^162^, ^206^, ^207^, ^208^
Cervical cancer	YAP, TAZ	Cisplatin	^209^
Chronic myeloid leukemia	YAP	Imatinib mesylate	^210^

EGFR‐TKIs, epidermal growth factor receptor tyrosine kinase inhibitors.

### 6.1 In respiratory tumors

Lung cancer is the leading cause of cancer‐related mortality globally.^211^ Small cell lung cancer (SCLC) is characterized by rapid tumor growth, extensive metastasis, chemotherapy resistance, and recurrence, while NSCLC is the most common type.^212^ The advent of tyrosinase inhibitors has substantially improved survival rates in lung cancer patients.^213^, ^214^ Unfortunately, most NSCLC patients develop resistance to the available tyrosine kinase inhibitors (TKIs).^215^ Recent research has focused on how Hippo signaling contributes to NSCLC drug resistance. It is known that YAP participates in the progression and metastasis of NSCLC.^216^, ^217^ YAP activation was found to cause erlotinib resistance in human NSCLC.^188^ Additionally, YAP is crucial to cancer resistance to EGFR‐TKIs by modulating the expression of PD‐L1 in NSCLC.^218^ In EGFR‐TKI‐acquired resistant NSCLC, targeting YAP therapy may become an attractive option.^219^ AXL, a member of the Tyro3‐Axl‐Mer receptor tyrosine kinase subfamily, has been identified as an essential downstream effector of YAP that contributes to the YAP‐driven resistance to EGFR‐TKIs. Combining inhibition of EGFR with the YAP/AXL axis would provide clinicians with a new strategy to combat drug resistance.^187^, ^189^ Furthermore, natural drugs may also influence tumor drug resistance by modulating Hippo signaling. For example, Rg3 is involved in mediating the chemosensitivity of Hippo signaling.^190^ Sophoroside activates Hippo signaling to enhance cisplatin sensitivity.^191^

Hippo pathway has rarely been studied in SCLC. Considering the heterogeneity of SCLC among individuals, further study of SCLC may help predict its efficacy and prognosis. In the clinical setting, SCLC in the YAP/TAZ subgroup could respond more effectively to chemotherapy or targeted therapy in combination with inhibitors of YAP/TAZ. Accordingly, recognizing this subgroup is essential from a prognostic and therapeutic perspective.^220^

### 6.2 In gastrointestinal tumors

Therapeutic resistance is a major challenge in GC treatment. The Hippo pathway has been proven to be a promising drug target to overcome this challenge. Furthermore, the Hippo pathway has a substantial impact on GC cells' malignant biological behavior.^221^ The mobilization or upregulation of YAP1 can lead to tumor occurrence and resistance to chemotherapeutic drugs such as mitomycin C, cisplatin, and etoposide in GC.^222^ Gastric cancer stem cells (CSCs) have a high YAP/TAZ‐TEAD molecular signature and transcriptional activity that maintains the characteristics of chemotherapy resistance and tumorigenicity.^223^ Targeting YAP1/TAZ‐TEAD transcriptional activity inhibits the tumorigenic properties of gastric CSCs. Notably, Paired box 6 (PAX6) induces palbociclib resistance in GC cells by promoting promoter hypermethylation of LATS2.^224^

In colorectal cancer patients, the oncogene YAP1 was significantly associated with an adverse prognosis and cetuximab resistanc.^192^ Moreover, YAP and RAR–RXR interaction promotes 5‐FU resistance in colorectal cancer cells by elevating the expression of stemness genes.^194^ Que et al. revealed that the downregulation of miR‐874‐3p resulted in inactivating Hippo signaling by targeting YAP and TAZ, which leads to the chemotherapy resistance of colorectal cancer to 5‐FU.^193^ Moreover, the abnormal expression of transcription factor AP‐2Y (TFAP2C) is related to the malignant progression of various tumors. TFAP2C inhibits Hippo signaling by upregulating ROCK1 and ROCK2 in colorectal cancer, thereby contributing to the stemness of colorectal cancer cells and the resistance to 5‐FU.^225^ Interestingly, studies have found that TAZ mediates tumor aggressiveness, dryness, and chemotherapy sensitivity in colon cancer. Targeting TAZ may be beneficial for colon cancer therapy in some cases.^226^

The Hippo pathway is one of the essential factors involved in HCC cell proliferation, metastasis, and chemoresistance.^227^ In HCC, YAP1 participates in drug resistance by inducing the expression of stemness markers and ATP‐binding cassette transporters.^195^ Furthermore, YAP contributes to sorafenib resistance via upregulating survivin in HCC.^196^ There is compelling evidence that ferroptosis is a significant contributor to tumorigenesis.^228^ For example, YAP/TAZ and ATF4 confer resistance to sorafenib in HCC through blocking ferroptosis.^229^

The YAP gene is usually abnormally activated in pancreatic cancer and can serve as a valuable biomarker.^230^, ^231^ It has been confirmed that a high level of YAP and TAZ is linked to worse outcomes in pancreatic cancer patients.^232^ Interestingly, catechol induced AMPK phosphorylation and decreased the expression of the Hippo pathway components, which enhanced the chemosensitivity of pancreatic cancer cells to gemcitabine.^198^ Taken together, these studies highlight the significance of Hippo signaling in overcoming drug resistance. Targeting the Hippo pathway may offer a promising avenue to overcome cancer drug resistance.

### 6.3 In urinary tumors

Renal cancer is the seventh most frequent cancer globally.^233^ Early‐stage renal cancer can be treated by active monitoring, ablation, or radical resection, but the results vary greatly. Thus, understanding why is imperative.^234^ There was a significant increase in the expression of YAP in clear cell renal cell carcinoma (ccRCC), which was associated with a poor prognosis for patients.^235^ Additionally, a study has shown that TEAD4 acts as a tumor‐promoting factor role in ccRCC and may impose a significant impact on its progression.^236^ Interestingly, Miao et al. found a close relationship between dihydrolipoamide branched transacetylase E2 (DBT) and the Hippo pathway in ccRCC. Specifically, membrane‐associated protein A2 (ANXA2) interacts with the lipoyl‐binding domain of DBT to trigger Hippo signaling, which reduces YAP nuclear accumulation and lipogenic genes transcription.^237^ There is evidence that overexpression of TAZ in renal cell carcinoma increases the sensitivity of cells to ferroptosis. Mechanistically, TAZ causes ferroptosis by upregulating the expression of epithelial membrane protein 1, which in turn induces the expression of nicotinamide adenine dinucleotide phosphate oxidase 4.^238^ This evidence suggests the therapeutic potential of TAZ in kidney cancer as well as other TAZ‐activated cancers.

Additionally, Hippo signaling has been demonstrated to be involved in the resistance to drugs in prostate cancer. CD44 plays a critical role in the progression and metastasis of docetaxel‐resistant prostate cancer cells through the Hippo–YAP pathway, which could represent a potential treatment target in docetaxel‐resistant prostate cancer.^199^ There is sufficient evidence showing that patients with high nuclear YAP1 in residual cancer after docetaxel‐based chemohormonal therapy have a higher recurrence rate than patients with low nuclear YAP1. Targeting the Hippo signaling pathway and steroid receptors may overcome the resistance to chemical hormones in prostate cancer.^239^ Decitabine treatment inhibits the expression of downstream oncogenic genes, restores RASSF1A, activates the Hippo signaling pathway, and enhances cisplatin and doxorubicin cytotoxicity. For bladder cancer, RASSF1A might thus be considered a therapeutic target for activating Hippo signaling.^200^

### 6.4 In reproductive tumors

YAP has been identified as a potential therapeutic target for multiple cancers,^53^, ^77^, ^188^, ^240^ yet its role in BC is undefined. The clinical evidence shows that TAZ/YAP contributes to the development of BC in multiple ways.^241^ USP9X facilitates the survival of cancer cells and confers resistance by activating YAP through protein stabilization.^205^ Ado‐trastuzumab emtansine (T‐DM1) has increased survival in HER2+ metastatic BC patients, but the emergence of drug resistance remains a challenge.^242^, ^243^ T‐DM1‐mediated HER‐2 inhibition increases ROR1 expression through YAP1 activation and confers drug resistance on tumor cells. Simultaneous inhibition of YAP1 and ROR1 in combination with conventional HER‐2 therapy may overcome treatment resistance problem.^203^ Evidence suggests that WW domain‐containing oxidoreductase inhibits YAP activity by increasing the phosphorylation of YAP in breast cance.^204^ The antipsychotic drug chlorpromazine inhibits BC cell stemness, downregulates Hippo–YAP signaling, and increases BC cell resistance to drugs.^244^ Moreover, methotrexate‐free can make BC stem cell lines and related tumors more sensitive to the drug resistance of transplanted tumors.^245^ It is reported that TEAD4 plays a tumor‐promoting role in BC.^246^ Furthermore, glucocorticoids can activate TEDA4 and promote BC stem cell survival, metastasis, and drug resistance. The development of TEAD4 inhibitors could help overcome drug resistance caused by glucocorticoids.^202^

It has been widely recognized that YAP1 mRNA levels are elevated in ovarian cancer.^20^ YAP may be an oncogene in OC, which usually induces drug resistance.^247^ The activation of YAP1 is closely related to poor prognosis, and sensitivity to paclitaxel has a significant predictive value in OC.^248^ It is reported that the expression of YAP and TEAD4 is positively correlated and the coexpression of YAP and TEAD4 is tightly relevant to the poor prognosis of ovarian cancer. Moreover, YAP/TEAD coactivators are involved in regulating cancer initiation and drug resistance.^206^, ^249^ Increasing research shows a significant role of lncRNAs in response to ovarian cancer treatment. For example, MiR‐149‐5p directly targets the key components MST1 and SAV1 of the Hippo signaling pathway, thus inactivating TEAD, making it a potential treatment target for ovarian cancer.^207^ A potential mechanism for LINC01508 targeting YAP and enhancing cisplatin sensitivity in ovarian cancer cells involves the inhibition of cell invasion, induction of apoptosis, and cell cycle arrest.^208^ Recently, researchers have found that the recurrence of chemotherapy resistance in patients with ovarian cancer is related to low TAZ expression and decreased sensitivity to ferroptosis. Interestingly, high expression of TAZ in ovarian cancer patients increases cells' susceptibility, whereas removing TAZ reduces cells' sensitivity to ferroptosis.^250^ Additionally, it has been shown that AJUBA enhances cisplatin resistance via Hippo signaling in cervical cancer. In patients with cisplatin‐resistant cervical cancer, AJUBA is overexpressed, linked to an unfavorable prognosis.^209^

### 6.5 In hematological malignancies

Although years of efforts have been made in treating hematological malignancies, drug resistance remains a significant challenge.^251^ In light of this, novel therapeutic strategies are essential. Recent research demonstrates that the Hippo signaling pathway effectors function as oncogenes in various cancers but act as tumor suppressors in hematological tumors.^252^, ^253^ In addition, some investigators have confirmed the potential function of YAP in drug resistance during targeted therapy.^254^ In patients with chronic myeloid leukemia (CML), long‐term exposure to imatinib mesylate (IM) upregulates miR‐181a and increases YAP levels, ultimately leading to resistance to IM.^210^ Li et al.^255^ found that YAP inhibitors may enhance the efficacy of IM treatment in patients suffering from CML. LATS2 expression in highly drug‐resistant leukemic cells has been proven to be significantly decreased and is associated with drug resistance and an unfavorable prognosis.^256^ Moreover, LATS2, AURKA, and AURKB are overexpressed in CML patients, and TAZ is overexpressed in CML IM‐resistant patients. Developing drugs targeting Hippo signaling and the Aurora kinase genes may offer a promising therapeutic option for treating chemotherapy‐resistant CML.^257^It is intriguing to find that BCR‐ABL may induce YAP tyrosine phosphorylation through Src family kinases, resulting in the expression of survivin and Cyclin D, which leads to leukemia in CML cells.^258^ Further research may provide novel ideas for the treatment of CML. Additionally, silencing YAP may also be a therapeutic strategy for acute promyelocytic leukemia, as it inhibits the proliferation of HL‐60 cells and induces apoptosis.^259^ In acute myeloid leukemia, miR‐7977 promotes the proliferation of bone marrow mesenchymal stem cells through inactivating Hippo signaling.^260^

Notably, the decrease of YAP1 expression can progress from normal plasma cells to multiple myeloma (MM).^252^ Numerous studies support aberrant activation of the Hippo pathway in DLBCL. Interestingly, targeting 14‐3‐3η inhibits tumorigenesis in DLBCL.^261^ Moreover, the downregulation of IGF‐1R expression in DLBCL can also reduce Hippo–YAP signaling activation, which offers novel therapeutic insights for DLBCL treatment.^78^

## 7 CONCLUSION AND FUTURE PERSPECTIVES

With a deeper understanding of Hippo signaling and the downstream mechanisms of YAP and TAZ, some key concepts concerning this pathway's role in cancer are becoming clearer. The well‐characterized functions of Hippo signaling are to control cell growth and organ size, usually acting as a complex tumor suppressor. It is widely accepted that dysregulation of this pathway leads to tumor invasion, metastasis, disease progression, as well as cancer drug resistance. Accordingly, therapeutic strategies targeting Hippo signaling could have favorable prospects for clinical translation.

However, emerging evidence indicates that YAP and TAZ may exhibit cancer‐promoting or cancer‐suppressing effects depending on cancer types.^14^ For example, high expression of YAP and TAZ is usually associated with poor prognosis in cancer patients, whereas high YAP and TAZ mRNA expression in hematological cancers or some solid tumors inhibits tumor growth.^252^, ^262^, ^263^ It may be more challenging to explore cancer therapies targeting the Hippo pathway. Moreover, Hippo signaling has been implicated in metabolic reprogramming and epigenetic regulation of tumors in a growing number of studies. These recurring themes present vulnerabilities that could be exploited in future cancer therapies. In‐depth mechanistic and functional investigations are required to effectively target the Hippo pathway for cancer treatment development. The rapid emergence and evolution of novel sequencing technologies may pave the wave for overcoming these challenges. Specifically, the integration of single‐cell genomic and transcriptomic analysis, as well as the integration of multiomics data, will allow us to gain a deeper comprehension of the function of the Hippo pathway in cancers to optimize cancer therapeutics.

Despite significant efforts to identify upstream signals of Hippo signaling, it remains unclear how these upstream signals are transmitted and how they are translated into coordinated physiological responses. Future therapies may benefit from a deeper understanding of these mechanisms. Drug resistance is one of the most challenging aspects of cancer treatment. Targeting the Hippo signaling pathway could be a promising therapeutic approach considering its significance in cancer drug resistance. Currently, there are no US FDA‐approved inhibitors of YAP/TAZ. However, compounds that inhibit YAP/TAZ indirectly or target substrates downstream of YAP/TAZ have risen to prominence for improving therapeutic response in the clinic. Further, BET inhibitors that block YAP/TAZ‐mediated transcription have proven useful in overcoming YAP/TAZ‐induced resistance. While protein kinase inhibitor therapies have been successful, there are concerns regarding the potential for inhibiting the Hippo signaling core kinases to contribute to cancer. TEAD palmitoylation inhibition blocks YAP–TEAD‐dependent transcription and is easily druggable by small molecules. This exciting finding provides an innovative perspective on targeting the Hippo pathway. However, considerable work is still needed to clarify clinical efficacy and toxicity. Further investigations are anticipated to confirm the safety, efficacy, patient stratification, and drug delivery of innovative Hippo pathway‐targeted interventions in cancer.

## AUTHOR CONTRIBUTIONS

L. L. wrote and edited this manuscript and created figures and tables. X. Z. and L. L. reviewed and revised the manuscript. X. Z. provided direction and guidance throughout the preparation of the manuscript. All authors read and approved the final manuscript.

## CONFLICT OF INTEREST STATEMENT

The authors have no relevant conflicts.

## ETHICS STATEMENT

Not applicable.

## Data Availability

Not applicable.
